# Effects of Low Stocking Densities on Zootechnical Parameters and Physiological Responses of Rainbow Trout (*Oncorhynchus mykiss*) Juveniles

**DOI:** 10.3390/biology10101040

**Published:** 2021-10-13

**Authors:** Jérôme Roy, Frederic Terrier, Michael Marchand, Alexandre Herman, Cécile Heraud, Anne Surget, Anthony Lanuque, Franck Sandres, Lucie Marandel

**Affiliations:** E2S UPPA, UMR1419 Nutrition Metabolism and Aquaculture, Aquapôle, Université de Pau et des Pays de l’Adour, INRAE, F-64310 Saint-Pée-sur-Nivelle, France; frederic.terrier@inrae.fr (F.T.); michael.marchand@inrae.fr (M.M.); alexandre.herman@inrae.fr (A.H.); cecile.heraud@inrae.fr (C.H.); anne.surget@inrae.fr (A.S.); Anthony.lanuque@inrae.fr (A.L.); franck.sandres@inrae.fr (F.S.); lucie.marandel@inrae.fr (L.M.)

**Keywords:** teleost fish, initial fish density, growth, survival, health status

## Abstract

**Simple Summary:**

Overall, this study has reported that lower stocking density significantly altered survival with several fish dying during the experiment and an alteration of growth and feed efficiency for the remaining fish. Concomitantly, our results showed that low stocking density induced a chronic stress altering the physiological responses of trout (parameters related to welfare, immune and inflammatory systems). Our results supported the hypothesis that a minimum number of fish is essential for culturing fish farmed in order to maintain healthy physiological responses allowing them an optimal growth.

**Abstract:**

The present study investigated the effect of low stocking density on growth, survival, feed parameters and physiological responses (blood metabolites, welfare indicators, immune biomarkers, and transcriptomic responses of stress and immune-related genes) on juvenile rainbow trout (*Oncorhynchus mykiss*) reared under a recirculating aquaculture system during 12 weeks. Fish (average weight 29.64 g) were reared in triplicate under four initial densities: nine fish per tank (D9, 3.76 ± 0.06 kg/m^3^), 18 fish per tank (D18, 7.66 ± 0.18 kg/m^3^), 27 fish per tank (D27, 9.67 ± 0.01 kg/m^3^) and 36 fish per tank (D36, 12.94 ± 0.14 kg/m^3^). Results showed that lower stocking density D9 significantly altered survival with several fish dying during the experiment and an alteration of growth and feed efficiency for the remaining fish. In parallel, the study revealed that low stocking density induced a chronic stress altering the physiological responses of trout by dysregulation of the inflammatory, immune system, and indolamine/catecholamine brain levels. In conclusion, regarding all the variables observed, low stocking density (D9) alters survival, growth and feed efficiency of rainbow trout with alteration of their physiological responses. Selecting appropriate fish density relating to rearing conditions proved to be an essential concern to improve welfare in an aquaculture context.

## 1. Introduction

The physiological responses of farmed fish are of major interest in aquaculture, as an understanding of these responses would allow optimization of feed efficiency and growth performance. Although the general aim of fish farmers is to produce healthy and good-quality fish, it is now accepted that these characteristics are closely linked to fish density. In the aquatic environment, high stocking density is a critical factor known to directly influence the growth and physiology of several teleost species [1,2,3,4,5], and is therefore one of the numerous concerns about fish welfare in the aquaculture context. In practice, the densities at which farmers keep their stock are based on experience, with codes of practice and handbooks being used as guide. This guidance specifies a 2–80 kg/m^3^ range, depending upon the holding system, stalling parameters (i.e., oxygenation, aeration, water flow), organic production, fish size and species. In practice, several countries typically operate within a density range of 15 to 40 kg/m^3^. Although 60 kg/m^3^ is considered as the maximum stocking density, even higher densities (100 kg/m^3^) have been achieved using intense aeration, oxygenation and high water flows. However, it has been demonstrated that rearing fish at inappropriate stocking densities may impair growth due to factors such as social interaction and the deterioration of water quality, which can affect both the feed intake and conversion efficiency of the fish [4]. An exposure to conditions out of the unconventional range (e.g., in stocking density) could be perceived as stress by fish [6], triggering a cascade of biological events to cope with these stressors, such as stress and welfare responses [7,8] or activations of physiological and behavioral responses [9] that impact fish performance (i.e., growth and reproduction) [4].

Among farmed fish, rainbow trout (*Oncorhynchus mykiss*) is the dominant freshwater salmonid farmed in Europe and North America. Although it can tolerate high stocking densities, in addition to obvious alterations in zootechnical parameters (growth, survival and feed efficiency) [4], overcrowding can negatively affect immune response [10] and antioxidant status [11], and increase the incidence of physical injury and disease development [12]. Authorities have suggested that densities of 30 to 40 kg/m^3^ are detrimental to rainbow trout welfare, although this view is not based upon scientific investigations. Based on scientific findings, several of the reviewed publications reported adverse effects of high densities on trout growth [4], with a recent study [13] explaining that rainbow trout stocked at 40 kg/m^3^ had a lower growth rate than trout stocked at the low density of 20 kg/m^3^. Nevertheless, in addition to discrepancies which exist with regard to maximum stocking density recommendations for rainbow trout [4] (including a lack of evidence that trout suffer from increased disease incidence, increased mortality or crowding stress), no evidence has made it possible to determine minimal stocking density or the optimal feed efficiency and growth performance of farmed rainbow trout. Although fish farming has the universal goals of achieving the highest density of fish without disturbing their welfare, and without compromising their growth and survival, this is not reflected in the related literature. Indeed, with the aim of respecting the “Three Rs’’ (replacement, reduction and refinement of animal use in research), farmed stocking densities cannot be applied in the research context.

Rearing the lowest possible stocking density of fish for conducting scientific research can have many advantages in terms of controlling reproduction, evaluating individual weight gain, and assessing the physiological responses of fish. However, compared to the mammalian model in research, salmonid fish such as rainbow trout form linear, dominance-based social hierarchies in both natural and artificial populations (e.g., in aquaculture systems) [14]. These social hierarchies depend on stocking densities and are essential throughout the life cycle of rainbow trout [15]. Indeed, since the end of 1930s, scientists have agreed that social hierarchies in fish groups are essential for an individual’s status [16], conferring many advantages including increasing growth rate and survival as well as decreased stress responses and improved welfare. Thus, it is therefore not conceivable to carry out scientific experiments on isolated trout.

Although numerous studies have examined the impact of high stocking density on various parameters (water quality, temperature, feeding, reproduction, welfare, wound) in various fish models [17,18,19,20], there are no studies investigating the effects of low initial stocking density (lower than 15 kg/m^3^) in hand-fed farmed fish on growth, feed parameters and physiological responses.

To achieve these objectives, we conducted our study over the course of twelve weeks in juvenile rainbow trout, evaluating the impact of four initial stocking densities: 9, 18, 27 and 36 initial fish per tank. At the end of the experiment, in addition to zootechnical parameters (growth, survival and feed parameters), we observed the physiological responses of fish by determining at the transcriptomic level the patterns of expression of sets of genes related to inflammation, stress and immunity response in blood tissue. In addition, plasma cortisol and lactate were quantified as indices of stress response, proportions of blood cells (erythrocytes and lymphocytes) were quantified as indices of immune response and dopamine (catecholamine) and serotonin (indolamine) pathways were investigated as indices of welfare.

## 2. Materials and Methods

### 2.1. Animal Handling

Experimentation was conducted according to the guiding principles for the use and care of laboratory animals for scientific research (Directive 2010/63/EU) and in accordance with the National Guidelines for Animal Care of the French Ministry of Research (decree n°2013-118, 1 February 2013). The experiment was carried out from May to August 2020 at the experimental facilities at Donzacq (permit number A40-228.1, Landes, France). The researchers in charge of the experiments received training and personal authorization delivered by French veterinary services.

### 2.2. Fish and Experimental Design

**Figure 1:**
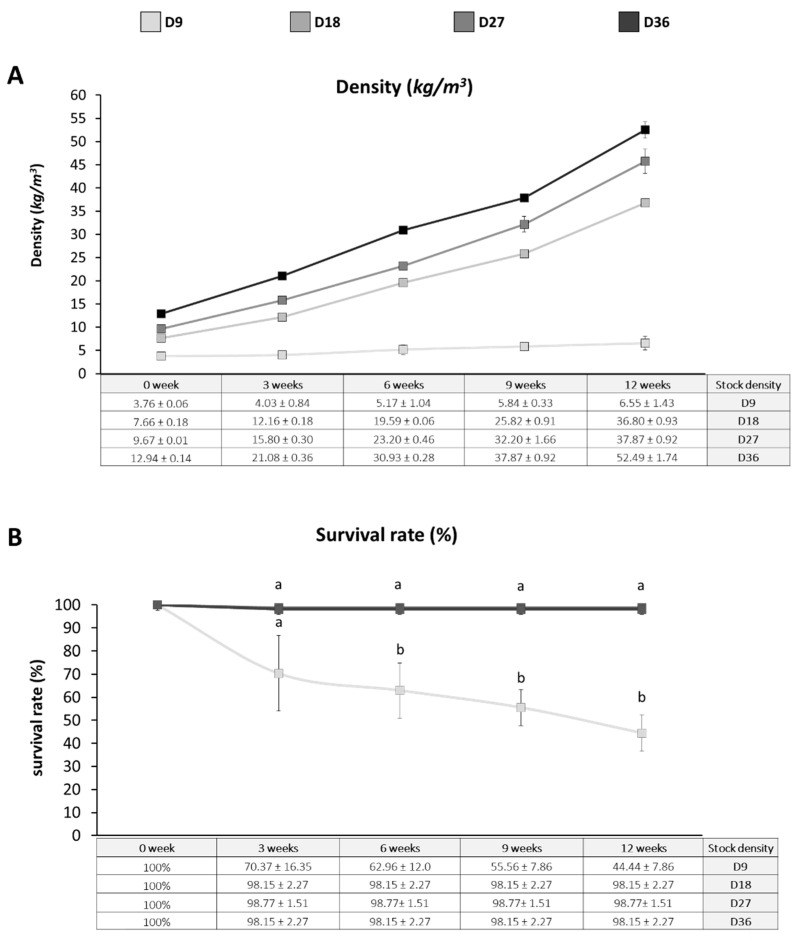
Density (**A**) and Survival rate (**B**) of rainbow trout hand-fed during 12 weeks with different stocking densities. (**A**) Density for each stocking density (D9, D18, D27, D36) is expressed in kg/m^3^ at each experimental timing period. (**B**) Fish survival is expressed as % ± S.E.M of survivors in relation to the initial number of fish in each tank, at each experimental timing period. Results are expressed in % of distribution as mean ± S.E.M (*n* = 3 tanks). One-way ANOVA, Tukey post hoc followed by a post hoc Tukey’s test in case of significant differences (*p* < 0.05, indicated with different letters. Mean values that do no share a common letter are significantly different).

The experiment was carried out during 12 weeks using rainbow trout originated from the same parental stock (INRAE Fish Farm of Lees-Athas, Permit number A64.104.1, vallée d’Aspe, France). Throughout the experiment, rainbow trout were reared at constant water temperature 18 °C in the INRAE experimental facilities at Donzacq, France under a natural photoperiod as previously described [21]. Juvenile trout (29.64 ± 0.37 g) were randomly distributed into 12 similar tanks sizes of 150 L (containing 70, 83 or 97 L volume depending of initial stocking density) at four initial stocking numbers (Figure 1A) each with three replicates: 9 fish per tank (D9, 3.76 ± 0.06 kg/m^3^), 18 fish per tank (D18, 7.66 ± 0.18 kg/m^3^), 27 fish per tank (D27, 9.67 ± 0.01 kg/m^3^) and 36 fish per tank (D36, 12.94 ± 0.14 kg/m^3^). During the experiment, all groups were fed a commercial dry pelleted feed from Skretting^®^ (France). The diet (digestible energy intake 23.75 kJ/g) contains 40% of crude protein; 21% of crude lipid; 7% of Ash. All fish were hand-fed twice a day to visual satiation with an interval of 8 h (at rate of 3–5% of body weight).

Each day, the amount of pellets distributed was measured for each tank. The weight of the remaining pellets was weighed and subtracted from the total weight measured before feeding. Throughout the trial, dead fish were removed daily and weighed (to identify dominant/dominated fish). Rates of fish survival were assessed as a percentage of the initial number of fish which survived. Every three weeks, fish were weighted and zootechnical parameters were calculated. No more weighing was done to avoid stress and loss in appetite that could result from handling. At the end of the trial, each fish sampled was weighted and measured. During the trial, water dissolved oxygen was minimum 7 mg/L, ammonia (total ammonia nitrogen) < 0.01 mg/L, nitrite < 0.04 mg/L and nitrate approximately 17 ppm. Water flow was 0.2 L/s per tank that renewed 6 times per h.

### 2.3. Zootechnical Variables and Analysis

At the end of 12 weeks of feeding, samplings were performed at 6 h after the last meal. Fifteen fish per condition (only the 12 remaining fish at the end of 12 weeks for D9 stocking density) were anesthetized with benzocaine (30 mg/L; Sigma-Aldrich^®^, Darmstad, Germany) and killed in a benzocaine bath at 60 mg/L (lethal dose recommended by the French ethics committee). Blood was removed from the caudal vein into EDTA syringes and rapidly centrifuged (3500 *g*, 15 min, 4 °C); the plasma and blood fluid recovered were then frozen and kept at −20 °C until analysis of the plasma metabolites. For smears of separated red and white cells samples, blood fluids were immediately washed with 1× PBS after separation and kept at 4 °C one day before performing blood smears. Brain and plasma samples for metabolites and gene transcriptional analysis were immediately frozen in liquid nitrogen and stored at −80 °C until analysis.

Variables related to growth were survival rate (% of initial number of fish per condition), density (kg/m^3^, total mass of tank/volume of tank) of initial number of fish per condition) final body weight (FBW), body weight gain (FBW minus initial body weight, IBW), daily growth coefficient (DGC, 100 × (FBW0.33 − IBW0.33)/days, % per day), specific growth rate (SGR, (ln(FBW) − ln(IBW) × 100)/t (in days)). Variables related to food intake were daily feed intake expressed in relative terms (% BW/day) and feed efficiency (FBW/food intake, FI). Daily digestive energy intake (DEI) was obtained by multiplying FI by the digestible energy (DE content) of the diet (estimated as 23.75 kJ g^−1^). To measure the relation between growth and welfare, we measured K coefficient of Fulton condition (fish weight/fish length^3^) which revealed the general physiological responses of the fish.

### 2.4. Gene Expression Measurement by Real-Time Quantitative PCR

Total RNA samples were analyzed on blood cells. Samples were extracted using TRIzol reagent (Invitrogen, Carlsbad, CA, USA), according to the manufacturer’s recommendations as previously described [22] and were quantified by spectrophotometry (absorbance at 260 nm) using a Nanodrop 1000 Spectrophotometer (LabTech, Heathfield, UK).

The integrity of the samples was assessed using agarose gel electrophoresis (1.5%). To avoid any contamination of gDNA, each sample was treated with the Turbo DNA Free Kit (Invitrogen) according to the manufacturer’s method and quantified again by spectrophotometry (absorbance at 260 nm).

One microgram of total RNA per sample was reverse transcribed into cDNA using the Super-Script III reverse transcriptase kit (Invitrogen) with random primers (Promega, Charbonnieres, France) according to the manufacturer’s instructions.

mRNA levels of cytokines and inflammation-related genes were determined by quantitative real-time qRT-PCR. Elongation factor-1α (*eef1α*) was considered as a reference gene. Each PCR product was systematically sequenced.

Quantitative RT–PCRs were carried out on a Light Cycle 480 II (Roche Diagnostics, Neuilly-sur-Seine, France) using SYBR Green I Master (Roche Diagnostics GmbH, Mannheim, Germany). PCR was performed using 2 µL of the diluted cDNA (40 times) mixed with 0.24 µL of each primer (10 µM), 3 µL of Light Cycle 480 SYBR Green I Master (Roche Diagnostics) and 0.52 µL of DNase/RNase free water (5 prime, Hamburg, Germany) in a total volume of 6 µL. The qPCR was initiated at 95 °C for 10 min, then followed by 45 cycles of a three-step amplification program (15 s at 95 °C, 10 s at 60 °C, 15 s at 72 °C). Melting curves were systematically monitored (5 s at 95 °C, 1 min at 65 °C, temperature gradient 0.11 °C/s from 65 to 97 °C) at the end of the last amplification cycle to confirm the specificity of the amplification reaction. Each PCR assay included replicate samples (duplicate of reverse transcription) and negative controls (reverse transcriptase and RNA free samples).

Relative quantification of target gene expression was performed using the E-method from the Light Cycler 480 software (v. SW 1.5; Roche Diagnostics). PCR efficiencies were measured by the slope of a standard curve using serial dilution of cDNA, and they ranged between 1.85 and 2.0. Transcripts were normalized to *ef1α* and exprimed as fold change vs. D9 density for all genes

### 2.5. Primer Design

**Scheme 1:**
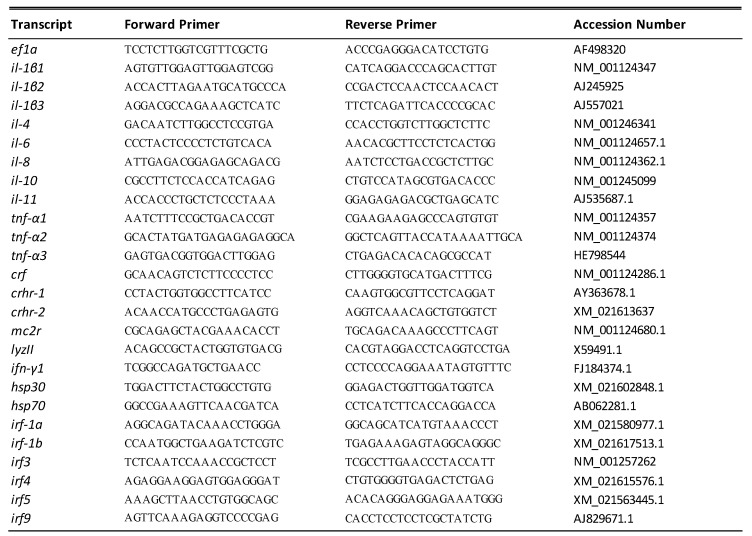
Nucleotide sequences of the PCR primers used to evaluate mRNA abundance of transcripts by RT-PCR.

The primer sequences used to amplify all genes and accession numbers of the primers are presented in Scheme 1. For gene targets that had not previously been validated, primers were tested on a pool of cDNA and amplified products were systematically sequenced.

The qPCR primers for interleukin: *il-1β* (1,2,3), *il-4, il-6, il-8, il-10 and il-11,* for tumor necrosis factor: *tnf-αa* (1,2,3), corticotropin releasing factor (*crf*), corticotropin-releasing hormone receptor *(crhr1, crhr2),* melanocortin 2 receptor *(mc2r),* lysozyme 2 *(lyzII*), interferon gamma (*ifn-γ1*), heat shock proteins (*hsp30, hsp70),* interferon regulatory factor *(irf-1a irf-1b, irf3, irf4, irf-5a, irf-5b* and *irf9)* were designed based on the conserved regions of the sequences in Gene Bank (Rainbow trout, Accession Number respectively). Multiple qPCR primer combinations were designed for each gene using Primer3 software [23] (Scheme 1). To optimize annealing temperature for the respective gene, PCR was performed with specific primer pairs and analysis of PCR products was done by electrophoresis on agarose gel (1.5%) [24]. Primers not reported in Figures were not expressed in red blood cells.

### 2.6. Cells Staining and Microscopy

Using standard light microscopy, smears of red blood cell and white blood cell samples were separated and stained with Giemsa. The purity of separated blood cells was manually examined by counting 10 fields of smears based on cell proportions. All photography was carried out using ImageJ Pro software.

### 2.7. Stress Biomarkers 

#### 2.7.1. Plasma Cortisol

The level of plasma cortisol was measured using a commercial kit (Immunotech, Marseille, France) following the manufacturer’s instructions based on a radio-immuno assay method that was previously described by White and Fletcher [25].

#### 2.7.2. Plasma Lactate

The plasma level of lactate was determined using the commercial Lactate Colorimetric Assay Kit II (BioVision Science Company, San Francisco, CA, USA) based on the manufacturer’s procedure.

#### 2.7.3. Plasma Lysozyme

The lysozyme activity of plasma was spectrophotometry analyzed based on the lysis of lyophilized particles of *Micrococcus lysodeikticus*, supported with the following reference [26,27].

### 2.8. Assay for Welfare Responses: Catecholamine and Indolamine Release

Catecholamines and indolamines were separated and detected using a Waters^®^ Acquity H-Class Plus UHPLC System equipped with a thermostatted autosampler supported with Waters^®^ Acquity Multi-λ Fluorescence Detector (Milford, MA, USA). Waters^®^ Empower™ Pro software was used for data acquisition and quantification.

Potassium dihydrogen phosphate (KH_2_PO_4_), potassium monohydrogen phosphate (K_2_HPO_4_), ethylenediaminetetraacetic acid and metaphosphoric acid (MPA); L-3,4-dihydroxyphenylalanine, homovanillic acid (HVA), serotonin and 5-hydroxyinddole-3-acetic acid (5-HIAA) were purchased from Sigma-Aldrich^®^ (Germany). All solvents used were MS grade.

Each brain was homogenized with Precellys^®^ (Bertin Technologies, Montigny-le-Bretonneux, France) tissue in a pH6.5 ± 0.05 20 mM phosphate buffer with 1 mM EDTA. Cellular debris was removed by centrifugation (20 min, 14,000× *g*, 4 °C). Proteins from the supernatant were precipitated with a volume-to-volume 10% (m/v) MPA solution and removed by centrifugation (5 min, 14,000× *g*, 4 °C). Finally, supernatant was filtered with a 0.22 µm PVDF filtration unit and stored at −20 °C until separation.

Chromatographic separation was performed with a Phenomenex^®^ (Torrance, CA, USA) PFP(2) column (150 × 4.6 mm, i.d. 3 μm). The column oven was set at 30 °C. Samples were maintained at 4 °C in the autosampler until injection. The injection volume was 10 μL. The flow rate was set at 0.4 mL/min.

A quaternary solvent system was developed with (A) pH4.3 ± 0.05 10 mM phosphate buffer, (B) methanol, (C) ultrapure water and (D) acetonitrile. The mobile phase was filtered through in-line 0.2 μm membrane filters. A linear gradient elution was achieved: 0–11 min: 80% A, 20% B; 16 min: 50% C, 50% D; 16–26 min: 50% C, 50% D; 27 min: 80% A, 20% B and 27–35 min (column equilibration): 80% A, 20% B. The eluate was monitored with a double excitation/emission fluorescence detection: 285/355 nm for 5-HT and 5-HIAA; 228/306 nm for L-DOPA and HVA.

Metabolites were identified comparing their RT to standard ones. Integration of peak areas was done to quantify metabolites. The samples were quantified against standard curves of at least 6 points run in triplicate. Standard curves were run at the beginning and the end of each chromatographic series. Quality control checks (blanks and standards) were run every 20 samples.

### 2.9. Statistical Analysis

All statistical analyses were performed with the R Software (version 3.6.1). All data are expressed as mean ± S.E.M. Treatment (stocking densities) effects were considered statistically significant at *p* < 0.05. When the normality of distributions was confirmed (Shapiro–Wilk’s and Levene’s tests, respectively), one-way ANOVAs were performed on the different variables obtained. When significant differences were demonstrated, means were compared using a Tukey’s post hoc analysis. Statistical difference was considered significant when the *p*-value was <0.05.

## 3. Results

### 3.1. Growth Performance

**Scheme 2:**
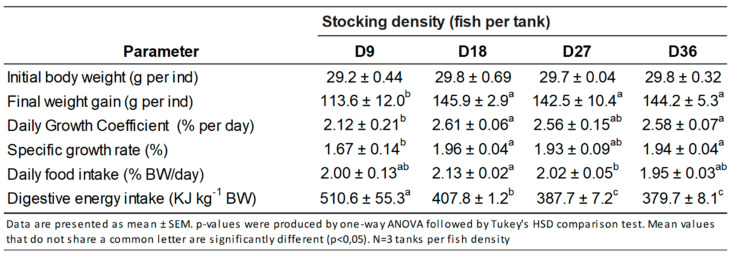
Effect of stocking densities on production parameters of hand-fed rainbow trout.

**Figure 2:**
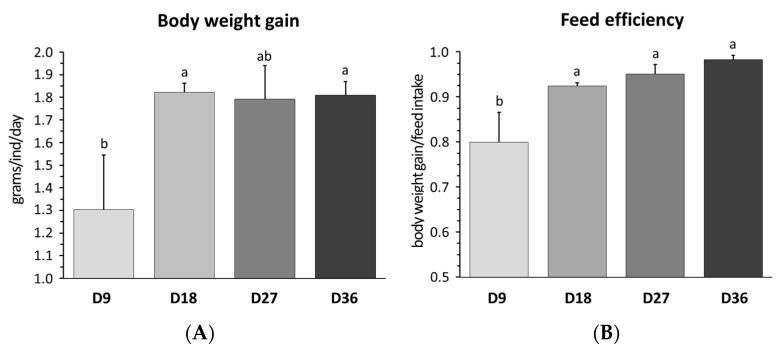
Body weight gain and Feed efficiency of rainbow trout hand-fed during 12 weeks with different stocking densities. (**A**) Representative body weight gain expressed in grams by individual at the end of the trial (12 weeks). (**B**) Representative feed efficiency expressed in body weight gain/feed intake at the end of the trial. One-way ANOVA followed by a post hoc Tukey’s test in case of significant differences (*p* < 0.05, indicated with different letters. Mean values that do no share a common letter are significantly different).

Effects of stocking densities on the evolution of fish density and survival rate are presented in Figure 1A,B, respectively; the production parameters, in Scheme 2; and the body weight gain (individually in Figure S1) and feed efficiency, in Figure 2. As shown in Figure 1A, stocking density increased during the experiment, resulting in maximum densities of 6.55 ± 1.43 kg/m^3^ for the D9 condition (fold increase of 1.74), 36.80 ± 0.93 kg/m^3^ for the D18 condition (fold increase of 4.81), 45.71 ± 2.62 kg/m^3^ for the D27 condition (fold increase of 4.72) and 52.49 ± 1.74 kg/m^3^ for the D36 condition (fold increase of 4.06) (Figure 1A). The survival rate was worst in the lowest stocking density condition (D9), reaching only 44.44% (Figure 1B). Survival rates were >98.15% in D18, D27 and D36 groups, with no significant differences among the stocking densities (Figure 1B).

FBW was significantly (*p* < 0.05) higher in D18, D27 and D36 conditions than in the lowest stocking density condition (D9) (Scheme 2), with higher heterogeneity between fish (Figure S1). DGC and SGR were significantly higher under D18 and D36 stocking densities compared to the D9 condition. Daily food intake was significantly higher for the D18 condition compared to the other conditions.

Body weight gain was significantly (*p* < 0.05) higher in D18 and D36 conditions than in the lowest stocking density (D9), with 1.30 ± 0.24 g/ind/day compared to more than 1.79 g/ind/day for all other stocking densities (Figure 2A). Feed efficiency was significantly (*p* < 0.05) higher in the three conditions with higher stocking density than in the lowest stocking density condition (D9) (Figure 2B).

### 3.2. Physiological Parameters

**Scheme 3:**
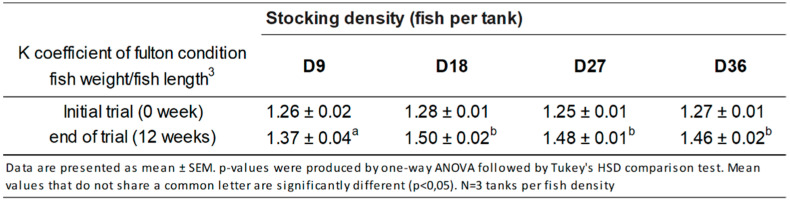
Effect of morphometry parameters (Fulton’s condition factor K) in different stocking densities on production parameters of hand-fed rainbow trout.

To investigate how stocking density affected the welfare and health status of fish, a morphometric parameter (Fulton’s K coefficient) was measured, and the results are presented in Scheme 3. After 12 weeks, K was significantly higher (*p* < 0.05) for the three highest stocking densities, with K > 1.46, compared to the D9 condition at 1.37 ± 0.04 (Scheme 3).

### 3.3. Blood Cell Type Proportions

**Scheme 4:**
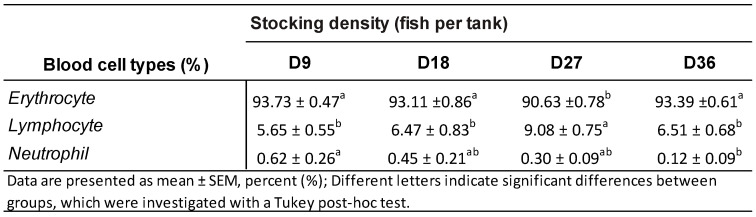
Proportion of blood cell types in rainbow trout between different fish stocking densities.

The erythrocyte count was significantly lower (*p* < 0.05) in the D27 condition compared to the three other conditions (Scheme 4). Lymphocytes were significantly lower (*p* < 0.05) under the two lowest stocking densities (D9 and D18) compared to the D27 and D36 conditions, whereas neutrophils were significantly lower (*p* < 0.05) for the D36 condition compared to the D9 condition (Scheme 4).

### 3.4. Lysozyme, Lactate and Cortisol Plasma Levels

**Figure 3:**
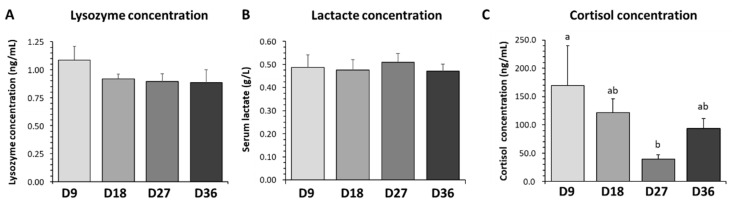
Lysozyme, lactate and cortisol plasma level of rainbow trout hand-fed during 12 weeks with different stocking densities. (**A**) Lysozyme (mg/mL, (**B**) Lactate (g/L) and (**C**) Cortisol level (g/L) in plasma of rainbow trout. Values are expressed as group mean ± SEM; one-way ANOVA, followed by a post hoc Tukey’s test in case of significant differences (*p* < 0.05, indicated with different letters. Mean values that do no share a common letter are significantly different).

Lysozyme (Figure 3A) and lactate (Figure 3B) levels showed no significant differences in the plasma of rainbow trout reared at various stocking densities. Regarding the cortisol plasma level (Figure 3C), fish reared at the lowest stocking density (D9) showed a significantly higher plasma cortisol concentration (*p* < 0.05) compared to those reared at the D27 stocking density.

### 3.5. Indolamine and Catecholamine Level Expression

**Figure 4:**
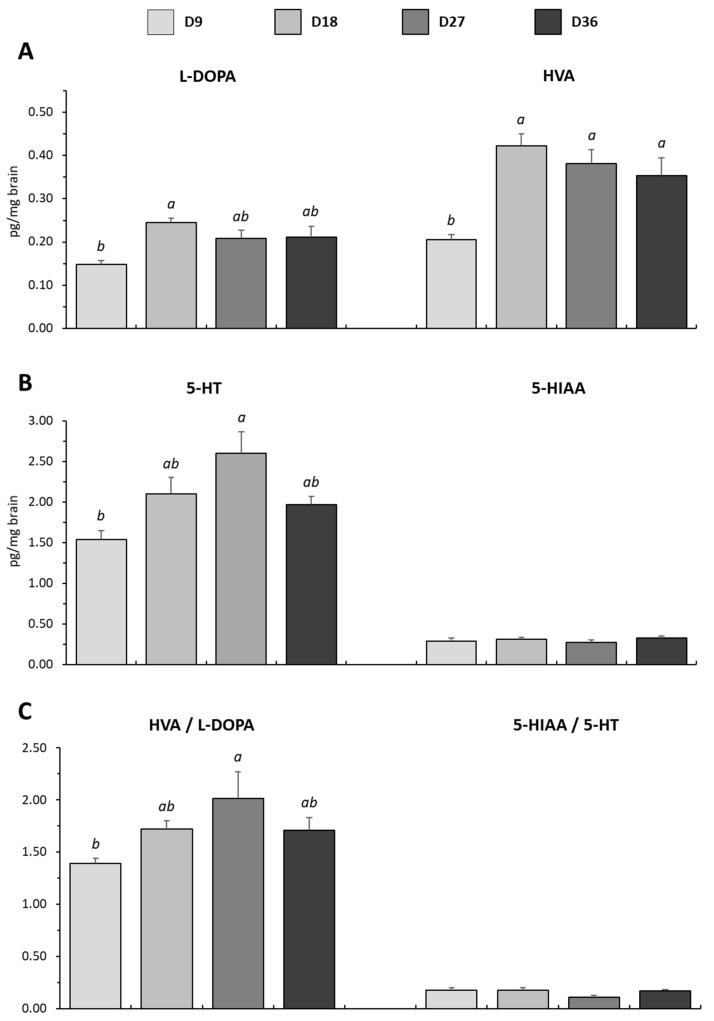
Brain concentrations of (**A**) catecholamines (L-DOPA, HVA), (**B**) indolamines (5-HT, 5-HIAA) and (**C**) corresponding monoamines pathway ratios HVA/L-DOPA and 5-HT/5HIAA in whole brain of rainbow trout hand-fed during 12 weeks with different stocking densities. Values are expressed as group mean ± SEM; one-way ANOVA, followed by a post hoc Tukey’s test in case of significant differences (*p* < 0.05, indicated with different letters. Mean values that do no share a common letter are significantly different).

Figure 4 shows the concentrations of the main markers of catecholamine (L-DOPA, HVA) and indolamine (5-HT, 5-HIAA) pathways and their corresponding ratios.

The brain turnover of catecholamines was the main factor affected by stocking density. Compared to the D18 stocking density, the level of L-DOPA (Figure 4A, left) was significantly lower in whole brain in rainbow trout reared at the lowest stocking density (D9). The level of HVA (Figure 4A, right) was also significantly lower in this group compared to fish reared at higher stocking densities (D18, D27 and D36). The HVA/L-DOPA ratios (Figure 4C, left) were significantly lower in the whole brains of fish reared at the lowest stocking density (D9) compared to those in the D27 group.

Regarding indolamines, the level of 5-HT was significantly lower for fish reared at the lowest stocking density (D9) compared to those in the D27 group (Figure 4B, left). The levels of 5-HIAA (Figure 3B, right) and the 5-HIAA/5-HT ratios (Figure 4C, right) were not affected by the different stocking densities.

### 3.6. Changes in mRNA Gene Transcript Expressions Related to Stress Response

**Figure 5:**
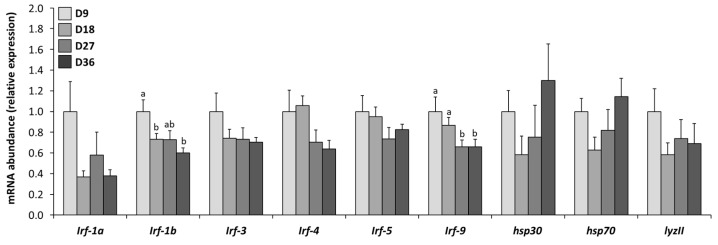
mRNA expression of transcripts related to immune markers and stress response in red blood cells of rainbow trout hand-fed during 12 weeks with different stocking densities. Relative gene expression measured by RT-PCR of *irf1, irf1B, irf3, irf4, irf5-1, irf5-2, irf9, hsp30, hsp70* and *lyzII* in red blood cells of rainbow trout. Values are expressed as group mean ± SEM; fold change vs. D9 density for all genes; one-way ANOVA, followed by a post hoc Tukey’s test in case of significant differences (*p* < 0.05, indicated with different letters. Mean values that do no share a common letter are significantly different).

The mRNA levels of immune-related genes in the red blood cells of rainbow trout were affected by the stocking density (Figure 5). Compared to fish reared at lower stocking density, fish reared at the stocking densities of D18 and D36 conditions showed a significant decrease in mRNA levels of *irf-1b* compared to D9 condition, and the fish reared at higher stocking densities (D27 and D36) showed significant density-dependent decreases in mRNA levels of *irf9*.

### 3.7. Changes in mRNA Gene Transcript Expressions Related to the Immune System

**Figure 6:**
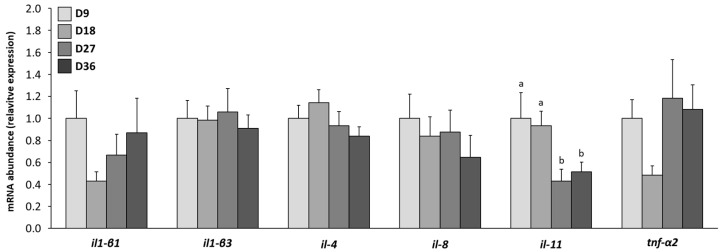
mRNA expression of transcripts related to inflammatory markers in red blood cells of rainbow trout hand-fed during 12 weeks with different stocking densities. Relative gene expression measured by RT-PCR of *il1-B1, il1-B3, il-4, il-8, il-11* and *tnf-α2* in red blood cells of rainbow trout. Values are expressed as group mean ± SEM; fold change vs. D9 density for all genes; one-way ANOVA followed by a post hoc Tukey’s test in case of significant differences (*p* < 0.05, indicated with different letters. Mean values that do no share a common letter are significantly different).

The effects of crowding on the expression of inflammation-related genes in rainbow trout are shown Figure 6. For the mRNA levels of *il-11*, fish reared at higher stocking densities (D27 and D36) showed a significant density-dependent decrease.

Other primers studied *(il1-b2, il-6, il-10, tnf-α1, tnf-α3, crf, crhr1, crhr2, mc2r, inf-γ1)* were not expressed in the red blood cells of rainbow trout.

## 4. Discussion

For the aquaculture industry, the stocking density is a factor of great importance, affecting fish health responses, causing health problems and affecting growth performance, ultimately leading to death when applied inappropriately for the cultured species. Many studies have evaluated the impact of high stocking density, but very few studies have evaluated the impact of low stocking density regarding the effects on the growth parameters, survival and health physiological responses of rainbow trout juveniles reared in aquaculture systems.

### 4.1. Effects of Low Stocking Density on Zootechnical Parameters of Trout

In this study, the main important result was that long-term low stocking density altered survival rates, with a mortality rate higher than 50% in the group with the lowest stocking density (D9). For the surviving fish in this group, alterations in growth and feed parameters associated with chronic stress were observed, as well as alterations in physiological responses. In this group, only nine fish were initially placed in each tank, obtaining a very low density below 4 kg/m^3^. At this density, this represents a space of 111 L/m^3^ of fish, which may have affected the establishment of a social hierarchy essential for their well-being. Indeed, it has been well-known for sixty years that salmonids form social hierarchies [28]. In environments in which resources such as food, shelter and mates are limited, social competition increases the dominance behaviors and fish strive to occupy the most profitable positions in the hierarchy. This behavior has been observed both in natural environments and in artificial populations such as aquaculture systems [14]. This social hierarchy is essential for individual fish, conferring many advantages such as increased growth rates and survival or decreased stress responses and improved welfare [16]. When confined to environments with limited resources, salmonid fish quickly establish linear dominance-based social hierarchies [29,30]. These stable hierarchies are thought to be beneficial to both dominant and non-dominant individuals because all fish experience reductions in the cost of serious fighting compared with those in unstable systems [31]. The establishment of a dominance hierarchy constitutes a social stress that can elicit a marked elevation of plasma cortisol in the subordinate fish [32]. This acute stress could enable the fish to access energy reserves but may be maladaptive during chronic stress owing to the suppression of immune function and continued mobilization of energy reserves [33]. Moreover, the cortisol concentrations will be significantly elevated during initial confinement. This social stress can be very severe, leading to the death of subordinate fish [34]. Some of these consequences were highlighted in our study, particularly our observation of higher plasma cortisol levels at the end of the trial for fish reared in the lower stocking density, suggesting chronic stress. However, no differences for secondary markers of stress such as lactate and lysozyme confirmed an acute stress induced by the trial.

Interestingly, in 2012, Sloman et al. revealed that a slight perturbation caused by increasing water flow altered the social behavior of brown trout and the physiological advantages of dominance [35]. Similarly, the same authors demonstrated that decreasing the water level had a disruptive effect on established social hierarchies [36]. The authors reported that these perturbations of increased water flow presumably increased the energy required by fish to maintain their position in the water column, thereby reducing the energy available for social interactions, which would disturb their physiological functions [36]. In our study, the establishment of a strong social hierarchy could be associated with a decreased number of fish reared at the lower stocking density. Regardless of initial stocking, our data suggest the establishment of a hierarchy by the presence of dominant fish (the biggest; Figure S1) in all 12 tanks. This suggests that the initial number of fish did not affect the establishment of a social hierarchy. However, the lower the initial number of fish, the greater the heterogeneity between the weights of fish after 12 weeks. Moreover, even larger fish died in the D9 group during the experiment, suggesting an alteration in their physiological responses. It seems that the lowest initial amount of fish did not prevent the establishment of a hierarchy, but affected the physiological responses of all fish in the group. It affected their stress level, causing a rapid alteration of their physiological responses (alteration of immune and inflammatory systems, leading to infections), and leading to mortality in some individuals [37].

Moreover, in this study, the low levels of catecholamines/indolamines observed in fish reared at lower stocking density provide support for the alteration of social behavior. Indeed, in mammals, brain serotoninergic and dopaminergic systems have been recognized as possible mediators of neuronal plasticity and cognitive functions, and are implicated in several behavioral mechanisms and processes, including stress effects and social behavior [38], assuming their activity as possible indications of “psychological” stress. Our data indicate that the dopamine precursors (L-DOPA) and serotonin (5-HT) levels in brain tissues were low for the D9 stocking density group compared to the other three groups. These results are in agreement with other studies showing changes of the brain monoaminergic turnover when fish confront a stressful condition [39,40,41,42]. We suggest that the reduction in growth performance was stress-induced, related to behavioral alterations and increased aggressiveness. The present changes in brain neurotransmitters for fish fed at lower density strongly reinforce this statement, suggesting probable changes in fish social interactions. More importantly, changes in social hierarchy and interactions, aggressive behavior and intra-species competition are undoubtedly related to changes in brain neurotransmitter activity, which in turn modulates the neuronal circuitry which mediates notably aggressive behavior [43].

Furthermore, feeding activity has been widely used as an observational indicator of the health status of fish, as stressors can decrease food intake or feed efficiency (metabolism), ultimately impacting the survival rate [44]. Our results confirm this observation, with a drastic alteration of feed efficiency but not food intake. It can therefore be assumed that the growth of rainbow trout is mainly influenced by stocking density, causing stress in the fish which ultimately alters their feed efficiency. Thus, the alteration in feed efficiency in rainbow trout is suggested to be secondary to stress/welfare responses, and it affected growth and survival rates.

### 4.2. Physiological Responses of Fish Were Altered by Long-Term Low Stocking Density

In addition to zootechnical parameters, our study reveals physiological effects in agreement with those observed in studies related to overcrowding. Indeed, many studies have observed that high density contributes to the suppression of immune responses, promoting bacterial infection and morphometric alteration, as was observed in our study. In parallel, transient elevations of circulating cortisol levels elicited by acute stressors are undoubtedly beneficial, but the long-term elevation of plasma cortisol as observed in this study for the lower stocking density group is associated with detrimental physiological consequences [8,45]. In 2009, Dhabhar et al. observed that chronic stress related to long-term overcrowding suppressed the innate and adaptive immune responses through neuroendocrine mechanisms [46]. Moreover, the influence of stocking density on the outbreak of infectious disease is a key factor in fish production [12]. There is also increasing evidence suggesting that cortisol directly and indirectly plays an important role in immune function (reviewed by Bly et al. [47]). All of the above argues for an adaptive role of cortisol during stress in fish. The results of the present study demonstrate that long-term low stocking density stimulated chronic stress responses, disrupted immune parameters (hematological indices) and modulated the expression of stress-, inflammatory- and immune-related genes in rainbow trout. Hematological parameters might provide useful information in assessing the pathologies and physiological responses of fish [48]. During stress, neutrophils play essential roles in the immune response and can be used to detect the initial situation in the fish’s immune system. After *Aeromonas hydrophila* bacterial infection, neutrophil concentrations increased in common carp (*Cyprinus carpio*) [49]. Our results revealed a higher proportion of neutrophils at lower stocking densities in rainbow trout, suggesting an alteration of immune system functioning. This higher proportion of neutrophils was accompanied by the lowest proportions of lymphocytes compared to the other stocking density groups. This observed difference in hematological parameters was also observed in a study related to overcrowding in juvenile stellate sturgeon [20]. In rainbow trout, the alteration of the immune system by overcrowding was also reported by Pottinger and Pickering [32]. When they transferred rainbow trout from their stock tank to smaller tanks at a range of stocking densities comprising individual fish, pairs of fish, five fish and ten fish per tank, it resulted in significantly elevated plasma cortisol levels and significantly reduced numbers of circulating lymphocytes, as observed for the D9 group in this study. Additionally, in the pairs of fish, one fish rapidly died from a presumptive bacterial infection and the surviving fish displayed high cortisol levels, its lymphocyte numbers remained depressed, and the fish lost weight throughout the experimental period. They concluded that these observations illustrated the importance and beneficial effect of the establishment of a social hierarchy in the relationships between fish in each pair. The results of our study support this conclusion, suggesting that low density led to the establishment of strong social hierarchy in rainbow trout leading to chronic stress and altered immune systems, leading to infections and death in some individuals.

In a study by Workenhe et al. evaluating the expression of immune-relevant genes, it was shown that red blood cells constitutively expressed toll-like receptors (TLRs) in rainbow trout [50]. TLR signaling plays important roles in various aspects of the innate immune response to pathogens. TLRs signal through the recruitment of specific adaptor molecules, which finally phosphorylate and translocate the IRF in the nucleus to transcribe IFN-α and -β and other IFN-induced genes. This family of IRFs is commonly used as a factor of immunity [51]. In our study, within the five monitored genes, *irf-1b* and *irf9* were upregulated in the D9 stocking density group, evidencing an alteration of the immune system for the fish in this group.

Additionally, chemokines like those in the interleukin family are involved in inflammatory reactions in fish and mediate immune responses after infection and injury [52]. However, there are no data on the effects of long-term low-stocking-density stress on the expression of cytokine genes in fish. The study of the mRNA levels of genes related to inflammatory markers in red blood cells revealed an up-regulation of interleukin-11 (*il-11*) for the fish in the D9 group despite no alterations in the regulation of other chemokines. The up-regulation of these markers (*irf1a, irf9* and *il-11*) was supported by the hematological results, with a higher proportion of neutrophils again suggesting an alteration of rainbow trout immune systems.

## 5. Conclusions

Overall, our findings indicate that the lowest evaluated stocking density (nine fish per tank; 3.76 kg/m^3^) was a chronic stressor for juvenile rainbow trout. This was revealed by an elevation of cortisol levels at the end of the trial, which led to negative effects on the physiological responses of the rainbow trout, affecting their survival rate as well as the growth and feeding behavior for surviving fish. These side effects may be related to alterations in social interactions between fish, leading to the disturbance of the established social hierarchy (harmful both for dominant and non-dominant fish). Additionally, this study suggests that the suitable minimal stocking density recommended for culturing rainbow trout juveniles is above 5 kg/m^3^, but must contain a minimum of 15–20 individuals. This density was shown to confer many advantages for individual status such as increased growth rates and survival rates with healthy physiological responses.

## Data Availability

All the data are available from the first author, and can be delivered if required.

## Supplementary Materials

The following are available online at www.mdpi.com/article/10.3390/biology10101040/s1, Figure S1: Individually final weight of rainbow trout reared with different initial stockings densities (D9, D18, D27, D36) of during 12 weeks. Values are expressed individually, for all fish in all tanks (T1, T2, T3), according to initial stocking density (D9, D18, D27, D36). Bold highlighted circle represent fish with the highest weight in the tank.
