# Supplementary material for: Effects of Low Stocking Densities on Zootechnical Parameters and Physiological Responses of Rainbow Trout (Oncorhynchus mykiss) Juveniles
Source: Biology (Basel). 2021 Oct 13;10(10):1040. doi: 10.3390/biology10101040 (PMC8533621; doi:10.3390/biology10101040)
Supplement: Supplementary file 1 [file biology-10-01040-s001.zip › biology-1375480-supplementary.pdf]

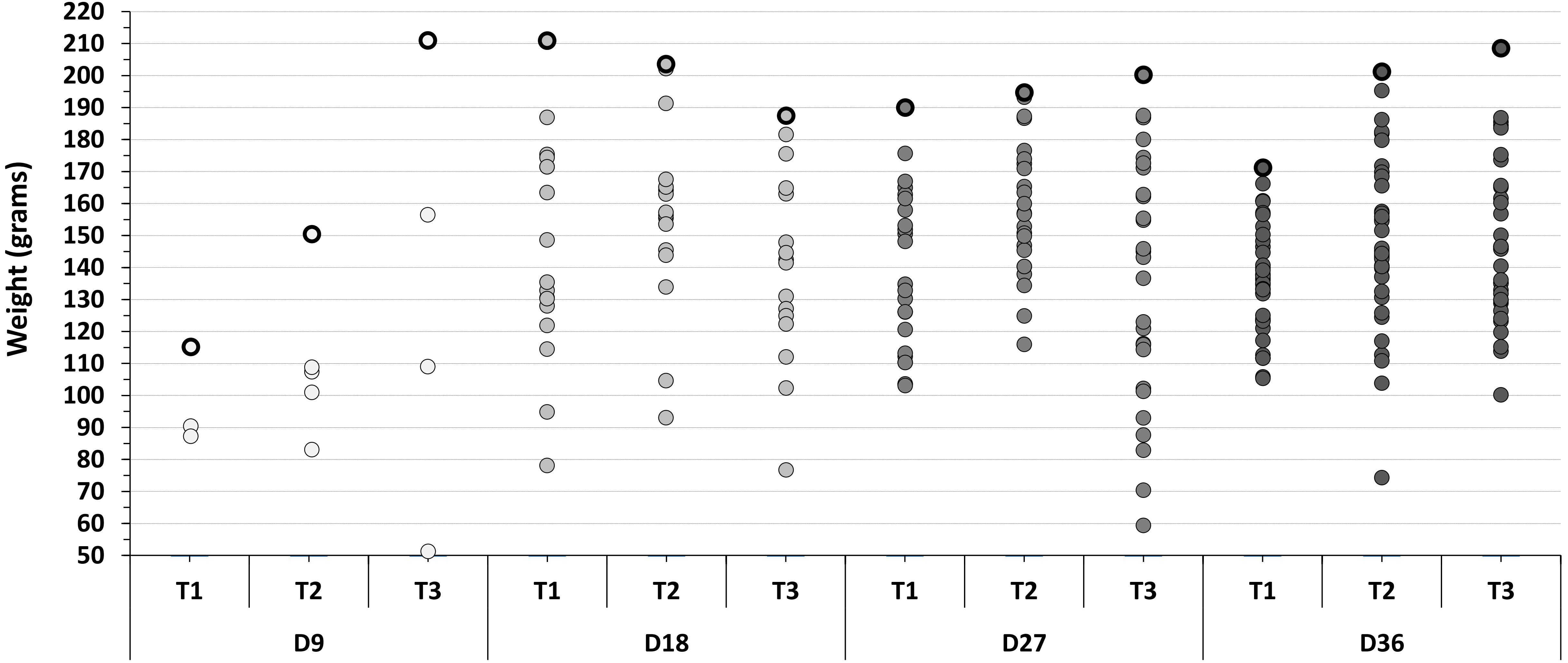

Figure S1 : Individually final weight of rainbow trout reared with different initial stockings densities (D9, D18, D27, D36) of during 12 weeks
